# Supplementary material for: Secondary Impact of COVID-19 Pandemic on People with Parkinson’s Disease—Results of a Polish Online Survey
Source: Brain Sci. 2021 Dec 26;12(1):26. doi: 10.3390/brainsci12010026 (PMC8774235; doi:10.3390/brainsci12010026)
Supplement: Supplementary file 1 [file brainsci-12-00026-s001.zip › Impact of the COVID in PD - the survey form - Supplementary Materials 1.pdf]

Secondary impact of the COVID-19 pandemic on people with Parkinson's disease

We present to you a set of questions concerning various aspects of life of a person with Parkinson's disease (PD) during the SARS-CoV-2 (COVID-19) coronavirus pandemic. After completing the form, you will be directed to a website where we provide you with a video with exercises for each day to do at home.

Please complete this short questionnaire (your answers will be anonymous). The study data will be published in the form of a research article. By submitting your answers, you consent to participation in the study.

At the end, there is also space for comments, remarks and any information that you would like to share with us. Thank you so much for your time!

The person completing the survey*

- Patient
- Caregiver

Do you feel particularly vulnerable to contracting SARS-CoV-2? *

- Yes
- No
- I do not know

Do you think that SARS-Cov-2 virus is particularly dangerous?

- Yes
- No

Why?

Have you tried to find information about any interactions between Parkinson's disease and COVID-19? *

- Yes
- No
- I do not know

If so, how were you looking for information on the interaction between PD and COVID-19? *

- Medical staff
- Internet
- Parkinson's Disease Patient Association / Foundation
- Family/Friends
- Not applicable
- Other:

Have you changed your behavior since the appearance of the new virus?

- Yes
- No
- Maybe

Do you undertake any preventive measures against COVID-19? *

- Yes
- No
- I do not know

If so, what are they? *

- Frequent hand washing
- Wearing a mask
- Using gloves
- Social distance
- Strict isolation
- Not applicable

Have you ever been in contact with someone who had COVID-19? *

- Yes
- No
- I do not know

Have you been diagnosed with SARS-CoV-2 infection? *

- Yes, I did have symptoms
- Yes, but I had no symptoms
- No
- No, but I had COVID-19 symptoms
- Other:

If so, did it affect the symptoms of Parkinson's disease? *

- Yes, they intensified
- Yes, they decreased
- No
- Other:

If so, which ones?

Are you afraid to go to the doctor or association for fear of infection? *

- Yes
- No
- Not anymore, but I had such a period during the pandemic

Have you had a problem with access to medications that are constantly taken? *

- Yes

- No

Have you had a problem with access to rehabilitation? *

- Yes
- No
- For some time during the pandemic I had a problem with it, but now I have returned to exercise
- Other:

During the restrictions, did you visit the website of an association or foundation for people with Parkinson's disease to find information about possible treatments and/or exercises? *

- Yes
- No
- I do not remember

During the restrictions, do you remain active in a different way, by exercising? *

- Yes
- No

If so, what are the activities? *

- Home training as before
- Individual exercises with a physiotherapist/instructor at home
- Individual exercises with an online physiotherapist/instructor
- Self-found exercises
- Walks
- Team/contact sports in small, trusted groups, e.g. table tennis, boxing
- Cycling
- Other:

How would you rate your activity before the pandemic? *

Little active 1 2 3 4 5 Very active – I exercised min. 5 times a week

How would you rate your activity NOW? *

Little active 1 2 3 4 5 Very active – I exercise min. 5 times a week

Do you think that the COVID-19 pandemic has affected your fitness? *

- Yes, negatively
- No, nothing has changed
- Yes, positively, I have motivated myself and I am feeling better
- Other:

Have you performed regular exercises to stimulate cognitive memory since the beginning of the pandemic? *

- Yes
- Not
- I do not know

Have you performed regular speech therapy exercises since the beginning of the pandemic? *

- Yes
- Not

Have you noticed the deterioration of your Parkinson's disease symptoms since the COVID-19 pandemic started? *

- Yes
- Not
- Maybe

If so, which symptoms have worsened *

- Motor slowdown
- Muscle stiffness
- Balance disorders
- Gait problems
- Problems with daily activities
- Problems with mood, motivation
- Problems with memory
- Depression
- Not applicable
- Other:

Have you felt anxious since the beginning of the pandemic? *

- Yes
- Not
- Maybe

Have you experienced a feeling of isolation since the beginning of the pandemic? *

- Yes
- Not
- Maybe

Has contact with friends/family deteriorated since the beginning of the pandemic? *

- Definitely yes, I am afraid of visiting people and I try not to leave the house
- Rather yes, fear of infection is less common
- Rather not, the same people visit/I go to the same places
- Definitely not, nothing has changed
- Another:

Self-perceived quality of life BEFORE the COVID-19 pandemic *

- Very good
- Okay
- Normal
- Bad
- Very bad

Self-perceived quality of life NOW during the COVID-19 pandemic *

- Very good
- Okay
- Normal
- Bad
- Very bad

Age *

Sex *

- Woman
- Man

Duration of illness *

- under 1 year
- 1-5
- 6-10
- 11-15
- over 15 years

Education *

- Primary
- Junior high school
- Basic - vocational
- Secondary
- Tertiary

Professional situation *

- Working – full-time work/self-employment
- Odd job
- Unemployed
- Pensioner

Domicile *

- Village
- Town up to 50,000 residents
- Town up to 150,000 residents
- City up to 500,000 residents

- City with over 500,000 residents

How do you rate your degree of independence in carrying out daily activities? *

- Complete independence
- A little help required
- Total dependence
- Unknown

Do you use amantadine (Viregyt K, Amantix)?

- Yes
- No
- I do not know

Do you have any comments, remarks?
