# Supplementary material for: Secondary Impact of COVID-19 Pandemic on People with Parkinson’s Disease—Results of a Polish Online Survey
Source: Brain Sci. 2021 Dec 26;12(1):26. doi: 10.3390/brainsci12010026 (PMC8774235; doi:10.3390/brainsci12010026)
Supplement: Supplementary file 1 [file brainsci-12-00026-s001.zip › Supplementary Materials 2- survey results.pdf]

**The results of the survey "Impact of Covid-19 pandemic on Parkinson's disease patients – results of a Polish online survey".**

The percentages presented do not have to sum up to 100 due to the rounding used.

The person completing the survey*

- Patient by themself *21%*
- With the help of their caregiver *79%*

Do you feel particularly vulnerable to contracting SARS-CoV-2? *

- Yes *55%*
- No *28%*
- I do not know *17%*

Do you think that SARS-Cov-2 virus is particularly dangerous?

- Yes *96%*
- No *4%*

Why? *Open question. The most frequent answers:*

| *Substantiation* | *No. of people* | *% of the total number of respondents [N = 47]* | *% convinced of the threat [n = 45]* | *% providing any substantiation* |
|---|---|---|---|---|
| ***High infectivity*** | *22* | *47%* | *49%* | *63%* |
| *Severe disease, complications* | *18* | *38%* | *40%* | *51%* |
| *High mortality* | *9* | *19%* | *20%* | *26%* |

Have you tried to find information about any interactions between Parkinson's disease and COVID-19? *

- Yes *43%*
- No *51%*
- I do not know *6%*

If so, how were you looking for information on the interaction between PD and COVID-19? *

- Medical staff *0%*
- Internet *53%*
- Parkinson's Disease Patient Association/Foundation *11%*
- Family/Friends *4%*
- Not applicable *32%*

Have you changed your behavior since the appearance of the new virus?

- Yes *89%*
- No *6%*
- Maybe *4%*

Do you undertake any preventive measures against COVID-19? *

- Yes *98%*
- No *2%*
- I do not know *0%*

If so, what are they? *

| ***Protection*** | ***Number of respondents*** | ***% of respondents [N=47]*** | ***% of them used the protection [n=46]*** |
|---|---|---|---|
| ***Wearing a mask*** | *46* | *98%* | *100%* |
| ***Frequent hand washing*** | *42* | *89%* | *91%* |
| ***Social distance*** | *37* | *79%* | *80%* |
| ***Using gloves*** | *18* | *38%* | *39%* |
| ***Rigorous closure*** | *10* | *21%* | *22%* |

Have you ever been in contact with someone who had COVID-19? *

- Yes *32%*
- No *51%*
- I do not know *17%*

Have you been diagnosed with SARS-CoV-2 infection? *

- Yes, I did have symptoms *4%*
- Yes, but I had no symptoms *0%*
- No *94%*
- No, but I had COVID-19 symptoms *2%*

If so, did it affect the symptoms of Parkinson's disease? *

- Yes, they intensified – *answered by one of two persons with confirmed diagnosis of SARS-CoV-2 infection*
- *No – answered by one of two persons with confirmed diagnosis of SARS-CoV-2 infection*

If so, which ones? *Tremor, stiffness, balance disorders and easy fatigability.*

Are you afraid to go to the doctor or association for fear of infection? *

- Yes *36%*
- No *34%*
- Not anymore, but I had such a period during the pandemic *30%*

Have you had a problem with access to medications that are constantly taken? *

- Yes *36%*
- No *64%*

Have you had a problem with access to rehabilitation? *

- Yes *45%*
- No *28%*
- For some time during the pandemic I had a problem with it, but now I have returned to exercise *11%*
- Other: not using *6%*, others *10%*

During the restrictions, did you visit the website of an association or foundation for people with Parkinson's disease to find information about possible treatments and/or exercises? *

- Yes *55%*
- No *32%*
- I do not remember *13%*

During the restrictions, do you remain active in a different way, by exercising? *

- Yes *94%*
- No 6%

If so, what are the activities? *

| *Type of activity* | *Number of respondents* | *% of respondents [N=47]* |
|---|---|---|
| ***Home training as before*** | *26* | *55%* |
| ***Walks*** | *25* | *53%* |
| ***Self-found exercises*** | *15* | *32%* |
| ***Individual exercises with a physiotherapist at home*** | *7* | *15%* |
| ***Individual exercises with physiotherapist online*** | *7* | *15%* |
| ***Cycling*** | *7* | *15%* |
| ***Team sports (e.g. boxing)*** | *6* | *13%* |
| ***Other activities*** | *6* | *13%* |
| ***None*** | *3* | *6%* |

How would you rate your activity before the pandemic? Mark the answer on a scale of 1 (little active) to 5 (very active – I exercised min. 5 times a week) *

- 1 *2%*
- 2 *13%*
- 3 47%
- 4 *23%*
- 5 *15%*

How would you rate your activity NOW? Mark the answer on a scale of 1 (little active) to 5 (very active – I exercise min. 5 times a week)*

- 1 *17%*
- 2 *34%*

- 3 28%
- 4 *15%*
- 5 *6%*

Do you think that the COVID-19 pandemic has affected your fitness? *

- Yes, negatively *70%*
- No, nothing has changed *30%*
- Yes, positively, I have motivated myself and I am feeling better 0%

Have you performed regular exercises to stimulate cognitive memory since the beginning of the pandemic? *

- Yes *32%*
- Not *60%*
- I do not know *9%*

Have you performed regular speech therapy exercises since the beginning of the pandemic? *

- Yes *19%*
- Not *81%*

Have you noticed the deterioration of your Parkinson's disease symptoms since the COVID-19 pandemic started? *

- Yes *55%*
- Not *17%*
- Maybe *28%*

If so, which symptoms have worsened *

| *Symptom* | *Number of respondents* | *% of people reporting deterioratig symptoms [n=39]* | *% of all respondents [N=47]* |
|---|---|---|---|
| ***Gait problems*** | *12* | *31%* | *26%* |
| ***Stiffness*** | *10* | *26%* | *21%* |
| ***Problems with daily activities*** | *10* | *26%* | *21%* |
| ***Slowness of movement (bradykinesia)*** | *9* | *23%* | *19%* |
| ***Problems with mood, motivation*** | *8* | *21%* | *17%* |
| ***Balance disorders*** | *6* | *15%* | *13%* |
| ***Problems with memory*** | *6* | *15%* | *13%* |
| ***Depression*** | *4* | *10%* | *9%* |
| ***None reported*** | *8* | *21%* | *17%* |

Have you felt anxious since the beginning of the pandemic? *

- Yes *38%*
- Not *34%*
- Maybe *28%*

Have you experienced a feeling of isolation since the beginning of the pandemic? *

- Yes *40%*
- Not *36%*
- Maybe *23%*

Has contact with friends/family deteriorated since the beginning of the pandemic? *

- Definitely yes, I am afraid of visiting people and I try not to leave the house *68%*
- Rather yes, fear of infection is less common *26%*
- Rather not, the same people visit/I go to the same places *2%*
- Definitely not, nothing has changed *4%*

Self-perceived quality of life BEFORE the COVID-19 pandemic *

- Very good *9%*
- Okay *32%*
- Normal *53%*
- Bad *6%*
- Very bad *0%*

Self-perceived quality of life NOW during the COVID-19 pandemic *

- Very good *2%*
- Okay *6%*
- Normal *34%*
- Bad *49%*
- Very bad *6%*

Do you use amantadine (Viregyt K, Amantix)?

- Yes *19%*
- No *66%*
- I do not know *13%*

The demographic data is presented in the manuscript in the Tab. 1.
